# The potential impact of adding genetic markers to clinical parameters in managing high-risk prostate cancer patients

**DOI:** 10.1186/2193-1801-2-444

**Published:** 2013-09-08

**Authors:** Maria Jesus Alvarez-Cubero, Luis Javier Martinez-Gonzalez, Fernando Vazquez-Alonso, Maria Saiz, Juan Carlos Alvarez, Jose Antonio Lorente, Jose Manuel Cozar

**Affiliations:** Laboratory of Genetic Identification, Legal Medicine and Toxicology Department, Facultad de Medicina, Universidad de Granada, Avda.de Madrid, 11, 18071 Granada, Spain; Center GENYO (Pfizer-University of Granada-Andalusian Government Center for Genomics and Oncological Research), Granada, Spain; Service of Urology, University Hospital Virgen de las Nieves, Granada, Spain

**Keywords:** Biomarkers, High risk, Prostate cancer, RNASEL gene, Staging, Treatment

## Abstract

**Purpose:**

High-risk prostate cancer is a potentially lethal disease that is increasing in the diagnosis of prostate cancer patients. Compared to other prostate cancer patients (medium or low risk), management, diagnosis and treatment are not as successful among high-risk patients. Because the genetic characterization of prostate cancer patients is increasing, we aimed to determine whether genetic information in one of the primary associated genes, such as RNASEL (2', 5'-oligoadenylate-dependent RNase L), could be used as a biomarker to improve the quality of life and treatment among high-risk patients. The main objective is to identify genetic variants of RNASEL that could be associated with high-risk prostate cancer to improve the clinical managing of these patients.

**Methods:**

A total of 231 prostate cancer patients were genotyped for 7 variants of RNASEL gene. Clinical information was obtained from medical examinations and genetic analysis (amplification and sequencing 7 variants of RNASEL gene) were performed by the researchers. Data were processed by statistical analysis (Chi square and logistic regression) using SPSS v.15.0.

**Results:**

Comparisons between genotypes and clinical characteristics of patients revealed that individuals with GG in D541E, AA in R462Q and AG in I97L in RNASEL gene were high-risk patients according to the European Urology Guidelines.

**Conclusions:**

Genotyping the RNASEL gene with routine diagnostic techniques could confer a more precise diagnosis of high-risk prostate cancer patients and increase the diagnostic accuracy above the current rate of 70% due to the relation between the genetic variants of RNASEL gene and the risk of this cancer.

**Electronic supplementary material:**

The online version of this article (doi:10.1186/2193-1801-2-444) contains supplementary material, which is available to authorized users.

## Introduction

Prostate cancer (PCa) is the most commonly diagnosed cancer among men worldwide, with almost one million new cases each year. However, the exact definition of high-risk prostate cancer remains unclear. A consensus on a clear definition is still needed, which currently translates into a lack of specific patient counseling and clear treatment management (Bastian et al. 2012). The actual risk stratification of prostate cancer is based on the probability of recurrence after local treatment. Some pre-treatment parameters have been analyzed as potential prognostic factors. PSA and Gleason score are considered as pre-treatment parameters that, when combined with clinical stage, are used to provide a more accurate prognosis of the results in these patients.

Prostate cancer is currently classified as stages T1 and T2 in located PCa and locally advanced prostate cancer is classified into stages T3-T4. Today, according to the EAU (European Association of Urology) and AUA (American Urology Association) guidelines, radical prostatectomy is a reasonable treatment option for selected PCa patients with cT3a disease, Gleason Score 8–10, or PSA > 20 (Bastian et al. 2012). However, a better knowledge of the natural history of the disease and developments in treatment options have resulted in more sophisticated risk stratification systems (Marciscano et al. 2012). It is clinically important to identify patients with high-risk PCa early on because they will benefit the most from curative therapy (Bastian et al. 2012). Currently, systemic therapy has a limited role in the treatment of localized prostate cancer, although adjuvant androgen deprivation therapy (ADT) has yielded significant improvement in disease-free survival for men with high-risk features treated with definitive radiation and a significant overall survival advantage for men with Gleason scores of 8 or higher (Dorff et al. 2011).

There are two primary types of treatment: watchful waiting and radiotherapy. However, neither carries 100% accuracy. Furthermore, no randomized studies have compared more intensive treatments, such as radiotherapy or surgery, with watchful treatment. A combination of radiotherapy with androgenic deprivation treatment over a short period is highly recommended based on the results of a randomized Phase III trial (D'Amico et al. 2008). Many different diagnostic methods are available and are widely used in high-risk patients, including local staging (T-stage), computed tomography (CT), magnetic resonance imaging (MRI), digital rectal examination (DRE), transrectal ultrasonography (TRUS) and 11C-choline positron emission tomography (PET). Unfortunately, none of these methods offers greater than 70% accuracy (Rinnab et al. 2007).

Currently, genetic, environmental and dietary factors are considered as the main components of PCa risk, playing large roles in the etiology of this cancer. Some specific SNPs in the involved genes, such as RNASEL at 1q24-25 (also known as Hereditary Prostate Cancer gene 1 (HPC1)), have been related to an increased risk of developing prostate cancer (Alvarez-Cubero et al. 2012; Agalliu et al. 2010; Meyer et al. 2010). The use of genetic information (as biomarkers) in combination with clinical details could be used by specialists to provide genetic counseling to these patients and adjust their treatment. The main goal of this article was to identify an alternative biomarker that could be used to identify patients with high-risk prostate cancer.

## Materials and methods

### Participant recruitment

Participants were recruited from the Urology Service of the University “Hospital Virgen de las Nieves”, Granada, Spain from 2007 to 2011.

### Ethics statement

This study complied with the Declaration of Helsinki. Informed consent was obtained from all subjects before they were enrolled in the study. The study and use of archive samples for this project were approved by the Ethics Committee of the University “Hospital Virgen de las Nieves,” Granada, Spain.

### Participants

A total of 231 patients (histopathologically confirmed after abnormal serum PSA findings) were enrolled. The patients’ clinical information was noted by a urologist, who also made annotations about important parameters for prostate cancer, such as PSA, local stage (T-score), Gleason score, and demographic information, such as age and place of birth (see Table 1).

**Table 1:** **Characteristics of the studied population**

	n = 231
**Mean (SD**^**1**^**) age (years)**	66.9 (7.74)
**Stage**	
**1**	n = 9 (3.90%)
**2**	n = 140 (60.60%)
**3**	n = 47 (20.35%)
**4**	n = 23 (9.96%)
**Missing**	n = 12 (5.02%)
**Gleason score**	
**2-6**	n =145 (62.77%)
**7**	n = 43 (18.61%)
**8-10**	n = 28 (12.12%)
**Missing**	n = 15 (6.27%)
**PSA levels (ng/ml)**	
**≤ 4.0**	n = 1 (0.42%)
**4.1-10**	n = 98 (42.42%)
**10.1-20**	n = 63 (27.27%)
**> 20**	n = 40 (17.32%)
**> 1,000**	n = 2 (0.84%)
**Missing**	n = 27 (11.69%)

*n* (number of patients), *SD* (standard deviation).

The mean participant age was approximately 66.9 (SD = 7.74) years. Demographic information was also collected to know that all the participants were unrelated Caucasian men. Due to the characteristics of this pathology, all of the participants were men.

### Mutation detection

Genomic DNA was extracted from blood samples using an organic extraction procedure with phenol/chloroform/isoamyl alcohol and proteinase K. In all participants, the RNASEL gene was amplified and sequenced with specific primers designed to cover the main variants of this gene that are related to prostate cancer development (R462Q, D541E, E262X, 471delAAAG, G265X, M1I and I97L). All detected mutations were confirmed independently in this study.

### Study design

Subjects were included as high-risk patients if they met the following indications of the European Association of Urology (EAU) Guidelines:

- Local stage with values ≥ T2c;
- Gleason score > 7; or
- PSA > 20 ng/ml.

The National Comprehensive Cancer Network (NCCN) includes patients with T3 stage, a Gleason score ranging from 8–10 or PSA values > 20 ng/ml. A total of 217 patients meet these criteria and were catalogued as high risk patients.

In our design we have included NCCN as the risk classification system for classify our data.

In the first steps of the analysis, we determined which of the seven analyzed variants of the RNASEL gene (R462Q, D541E, E262X, 471delAAAG, G265X, M1I and I97L) differed between subjects (low and intermediate-risk PCa patients (control group) and high-risk PCa patients (case group)) and could be used as biomarkers for high-risk patients. We then conducted a case-case analysis among high-risk PCa patients to establish which of the variants associated with prostate cancer risk were also associated with a high-risk phenotype.

### Statistical analysis

Allele frequencies were calculated by gene counting method for all of the studied SNPs. Variants E262X, 471delAAAG, G265X and M1I were not further considered for statistical analysis due to the presence of only one genotype in the entire patient population. For each single nucleotide polymorphism (SNP), including R462Q (rs486907), D541E (rs627928) and I97L (rs56250729), the allele frequencies were compared using the *χ*^2^ test in SPSS v.15.0 (IBM SPSS Statistics 2011). The Hardy-Weinberg equilibrium test and an analysis of the linkage of the loci of the RNASEL gene were performed with ARLEQUIN v.3.5 software (Excoffier & Lischer 2010). Comparisons between each locus and clinical information, such as PSA (≤ 4.0, 4.1-10, 10.1-20, > 20, > 1,000 ng/ml), age (≤ 55, 56–60, 61–65, > 65 years), stage (1, 2, 3, 4) and Gleason score (2–6, 7, 8–10), were obtained with contingency tables using the *χ*^2^ test, the Monte Carlo test and Fisher’s exact test; an ANOVA was also performed with the data. All tests considered the nominal statistical significance (p-value) to be <0.05. To evaluate correlations between high-risk patients and genetic characteristics, a *χ*^2^ test was first performed among high-risk patients (Gleason score > 7, PSA > 20 ng/ml or T-stage > 2). An adjustment for each genotype was then conducted for AA and AG in R462Q and GG and TG in D541E using a logistic regression analysis.

## Results

**Figure 1:**
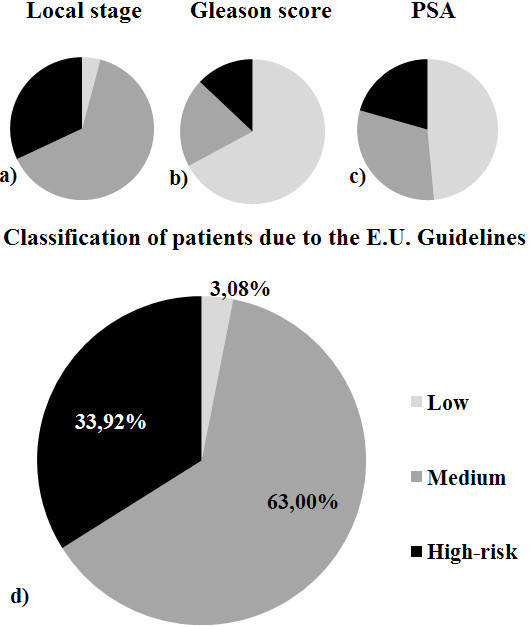
**Distribution of patients in three risk groups. a)** According to local stage; **b)** According to gleason score; **c)** According to PSA levels; and **d)** According to E.U. A Guidelines and meeting all three criteria together.

We could have included patients with a Gleason score above 7 (12.13%), a stage above 2 (30.54%) or a PSA value > 20 ng/ml (18.41%) as high-risk patients. Any of these single clinical factors could place a patient in a high-risk group, but this would have increased the percentage of individuals in this group (93.93%) (Figure 1). A more in-depth analysis was performed among the genotypes and all patients’ clinical characteristics (Table 2).

**Table 2:** **Descriptive statistical analyses of the patients and the clinical parameters with the RNASEL gene SNPs (R462Q, D541E and I97L)**

SNP		Age (years)	PSA values	Gleason score	T- stage
		median (SD)	median (SD)	median (SD)	median (SD)
**R462Q-rs486907**	AA	65.5 (8.64)	16 (429.92)	6 (1.63)	3 (0.78)
	GA	67 (7.75)	10.01 (201.81)	6 (1.25)	2 (0.68)
	GG	67 (7.25)	9.6 (15.44)	6 (1.07)	2 (0.56)
**D541E-rs627928**	GG	68 (8.26)	12.2 (388.05)	6 (1.44)	3 (082)
	TG	67 (7.84)	10 (20.25)	6 (1.21)	2 (0.67)
	TT	67 (6.19)	10 (6.57)	6 (0.95)	2 (0.35)
**I97 L-rs56250729**	TT	67 (7.83)	10.35 (157.94)	6 (1.29)	2 (0.71)
	GT	65	6.43	6	2
	GG	70	9.49	7	2

*SD* (standard deviation).

We examined the genotypes that could indicate high-risk PCa to include them as biomarkers with the clinical parameters. Only R462Q, D541E and I97L presented different genotypes among the patient population. The remaining SNPs (E262X, 471delAAAG, G265X, and M1I) could not be included in the analysis because only one genotype was identified among all of the patients.

Among the variants with statistically significant values, the genotypes associated with the worst prognoses and included in the recruitment for high-risk patients were GG in D541E, AA in R462Q and TG in I97L. The SNP distribution is summarized in Table 3. However, the variants that were associated with better clinical (Gleason score ≤ 7; PSA values < 20 ng/ml; T-stage < 3) characteristics were TT in D541E and GG in R462Q. We analyzed the clinical parameters among all of the patients, between the SNPs and clinical parameters, and most of the results were statistically significant (Additional file 1: Table S1). In high-risk patients, the p-values were statistically significant in R462Q and D541E (p ≤ 0.001) but not in I97L (p = 0.565) (Table 3). After correction using a logistic regression analysis, the TT and GG genotypes in the D541E and R462Q variants lost significance. However, the high-risk phenotypes AA in R462Q (p = 0.003) and GG in D541E (p = 0.017) were statistically confirmed (Table 3).

**Table 3:** **The distribution (%) of high-risk patients among the different SNP genotypes (R462Q and D541E) of the RNASEL gene,*X***^**2**^**statistical p-values**

SNP	Genotype	T stage		PSA		Gleason score > 7% (n)	P-value^1^	P-values^2^	OR (95% CI)
		Stage 3% (n)	Stage 4% (n)	> 20 ng/ml% (n)	> 1000 ng/ml% (n)				
**R462Q-rs486907**	AA	47.2 (17)	33.3 (12)	45.4 (15)	3.03 (1)	33.3 (12)	≤ 0.001	0.003	0.161 (0.048-0.543)
	GA	13.8 (16)	8.6 (10)	14.6 (16)	0.9 (1)	8.3 (10)		0.271	1.511 (0.724-3.153)
	GG	21.3 (16)	2.7 (2)	15.9 (11)	0.0 (0)	6.8 (5)		-	-
**D541E-rs627928**	GG	35.1 (26)	20.3 (15)	29.0 (20)	2.9 (2)	21.6 (16)	≤ 0.001	0.017	0.205 (0.056-0.755)
	TG	16.7 (20)	7.5 (9)	16.5 (18)	0.0 (0)	9.4 (11)		0.081	0.340 (0.101-1.142)
	TT	9.1 (3)	0.0	12.1 (4)	0.0 (0)	0.0 (0)		-	-

p-value^1^: derived from Pearson chi-square test.

p-value^2^: adjusted for high-risk patients by logistic regression.

OR: odds ratio.

CI: Confidence Interval.

## Discussion

Currently, the diagnosis of high-risk prostate cancer has approximately 70% accuracy. A digital rectal examination often underestimates the tumor extent; a positive correlation between the results of digital rectal examinations and pathological tumor stage was found in fewer than 50% of cases. The most commonly used method for viewing the prostate is transrectal ultrasound. However, only 60% of tumors are visible with transrectal ultrasound, and it was no more accurate at predicting organ-confined disease than digital rectal examination (Smith et al. 1997). Both computed tomography (CT) and magnetic resonance imaging (MRI) are now of a high technical standard, but neither modality is sufficiently reliable to make their use mandatory in the assessment of local tumor invasion (Jager et al. 2000). Endorectal MRI (e-MRI) may allow for more accurate local staging by complementing the existing clinical variables by improving the spatial characterization of the prostatic zonal anatomy and molecular changes (Masterson & Touijer 2008). Image quality and localization improves significantly with e-MRI compared with external coil MRI (Mullerad et al. 2005). The overall accuracy of 11C-choline positron emission tomography (PET) in defining local tumor stage (pT2 and pT3a-4) has been reported to be approximately 70%. The treatment options that are currently available for locally advanced prostate cancer are watchful waiting and radiotherapy. Watch waiting might be a treatment option for selected patients with non-poorly differentiated T3 tumours and a life expectancy of less than 10 years. Concomitant and adjuvant hormonal therapy for a total duration of 2–3 years, with external beam irradiation is recommended because it improves overall survival. Radical prostatectomy is optional in selected patients, in the context of multimodality treatment.

Many genes have been studied in order to determine a marker to be used as a diagnosis and prognosis factor among patients with prostate cancer. Among all the genes described as relevant in the developing of prostate cancer, the RNASEL gene has been highlighted as having the greatest effect (Alvarez-Cubero et al. 2012; Meyer et al. 2010). It has been recently determined that RNASEL gene variants D541E, R462Q and I97L are relevant mutations in the prognosis of the cancer (Alvarez-Cubero et al. 2012; Shook et al. 2007). Through the analysis of these variants and its correlation with clinical characteristics, it has been identified that genotypes with a worst prognoses (associated to individual who present clinical characteristics classified as high risk) were GG in D541E and AA in R462Q, whereas the ones that represented better clinical characteristics were TT in D541E, GG in R462Q and AC in I97L. A similar study in R462Q has been carried out in other populations, such as in Cleveland, Ohio and Detroit, Michigan, where men who are heterozygous with respect to the mutated allele were found to have a 50% greater risk of prostate cancer than non-carriers, and homozygotes had more than double the risk (Casey et al. 2002). Good results have been obtained with RNASEL gene but, as prostate cancer is a polygenic cancer, many other genes have to be analyzed and that will confer accurate information.

Even though genetic markers have been related to prostate cancer long time ago; none of them are currently used in clinical diagnosis. Though clinical diagnosis has not higher accuracy of 70%, these types of diagnosis or prognosis factors are not still used as a routine. The combination of anatomopathological, radiological techniques and genetic testing of genes related to prostate cancer will help clinicians both to better diagnose the type of cancer in each patient and to give a more specific treatment to them. That is why; we suggest the use of three SNPs in RNASEL gene as a combination factor with the present clinical sources.

## Conclusions

If the genetic information obtained in this study were added to the current diagnostic criteria, we could offer more specific treatment and increase the diagnostic accuracy above the current rate of 70%. However, we are conscious of the limited number of patients in this analysis, we suggest to make a deeper study by increasing the number of patients in this and others genes that have been already related to prostate cancer diagnosis and prognosis, to have a more accurate confirmation of the results. Both genetic and clinical data could confer relevant information about the control of prostate cancer. As mentioned in the results, individuals with GG in D541E, AA in R462Q and AG in I97L, in the RNASEL gene are classified as high-risk patients. Therefore, including RNASEL genotyping as an analytic parameter could confer a more accurate diagnosis in high-risk prostate cancer patients.

## Electronic supplementary material

Additional file 1: Table S1: X^2^ statistical p-values for all of the patients (SNPs: R462Q, D541E and I97L of the RNASEL gene). (DOC 31 KB)

## Abbreviations

ADT: Androgen deprivation therapy
AUA: American Urology Association
CT: Computed tomography
DRE: Digital rectal examination
EAU: European Association of Urology
EORTC: The Organization for Research and Treatment of Cancer
Gy: Gray
MRI: Magnetic resonance imaging
NCCN: National Comprehensive Cancer Network
TRUS: Transrectal ultrasonography
PCa: Prostate cancer
PET: and 11C-choline positron emission tomography
RNASEL: 2', 5'-Oligoadenylate-dependent RNase L
SD: Standard deviation.
