# Supplementary material for: The potential impact of adding genetic markers to clinical parameters in managing high-risk prostate cancer patients
Source: Springerplus. 2013 Sep 8;2:444. doi: 10.1186/2193-1801-2-444 (PMC3773101; doi:10.1186/2193-1801-2-444)
Supplement: Supplementary file 1 — Additional file 1: Table S1: X2 statistical p-values for all of the patients (SNPs: R462Q, D541E and I97L of the RNASEL gene). (DOC 31 KB) [file 40064_2013_503_MOESM1_ESM.doc]

**Table S1. X^2^ statistical p-values for all of the patients (SNPs: R462Q, D541E and I97L of the RNASEL gene).**

| **Patients** | **Age** | **PSA (ng/ml)** | **T-Stage** | **Gleason Score** |
| --- | --- | --- | --- | --- |
| **R462Q -rs486907** | 0.986 | 0.001 | 0.021 | 0.008 |
| **D541E-rs627928** | 0.047 | ≤0.001 | 0.006 | 0.001 |
| **I97L-rs56250729** | n.a | 0.002 | n.a | n.a |

n.a.: not applicable.
